# Correlation of Fine Needle Aspiration Cytology with Histopathology in the Diagnosis of Solitary Thyroid Nodule

**DOI:** 10.4061/2010/379051

**Published:** 2010-04-18

**Authors:** Manoj Gupta, Savita Gupta, Ved Bhushan Gupta

**Affiliations:** Department of General Surgery, Government Medical College, Jammu (J&K) 180005, India

## Abstract

*Background*. Fine needle aspiration cytology is considered the gold standard diagnostic test for the diagnosis of thyroid nodules. Fine needle aspiration cytology is a cost effective procedure that provides specific diagnosis rapidly with minimal complications. Based on the cytology findings, patients can be followed in cases of benign diagnosis and subjected to surgery in cases of malignant diagnosis thereby decreasing the rate of unnecessary surgery. Purpose of the present study was to correlate the fine needle aspiration cytology findings with histopathology of excised specimens. *Material and Methods*. This was a prospective study conducted on 75 consecutive patients between January 2003 and December 2005. All patients with clinically diagnosed solitary thyroid nodule who were clinically and biochemically euthyroid were included for study. Patients with multinodular goitre and who were hypothyroid or hyperthyroid were excluded from the study. *Results*. The sensitivity, specificity, accuracy, false positive rate, false negative rate, positive predictive value, and negative predictive value of FNAC for the diagnosis of neoplastic solitary thyroid nodules were 80%, 86.6%, 13.3%, 20%, 80%, and 86.6%, respectively. Commonest malignancy detected was papillary carcinoma in 12 patients. *Conclusions*. Fine needle aspiration cytology is a simple, easy to perform, cost effective, and easily repeated procedure for the diagnosis of thyroid cancer. It is recommended as the first line investigation for the diagnosis of solitary thyroid nodule.

## 1. Introduction

Solitary thyroid nodule is defined clinically as the localised thyroid enlargement with apparently normal rest of the gland. Solitary thyroid nodule is a common entity. Majority of these nodules are benign. The main goal of evaluating these nodules is to identify nodules with malignant potential.

A multitude of diagnostic tests like ultrasound, thyroid nuclear scan, and fine needle aspiration cytology (FNAC) is available to the clinician for evaluation of thyroid nodule. FNAC is considered the gold standard diagnostic test in the evaluation of a thyroid nodule, and other tests like ultrasound and nuclear scan should be used in conjunction with FNAC.

FNAC is simple, cost effective, readily repeated, and quick to perform procedure in the outpatient department with excellent patient compliance. Important factor for the satisfactory test includes representative specimen from the nodule and an experienced cytologist to interpret findings. It is often used as the initial screening test for diagnosis of thyroid nodules [1].

The prevalence of thyroid nodules ranges from 4% to10% in the general adult population and from 0.2% to 1.2% in children [2]. The majority of clinically diagnosed thyroid nodules are nonneoplastic; only 5%–30% are malignant and require surgical intervention [3].

FNAC is, however, not without limitations; accuracy is lower in suspicious cytology and in follicular neoplasms. The main aim of FNAC is to identify nodules that require surgery and those benign nodules that can be observed clinically and decrease the overall thyroidectomy rate in patients with benign diseases. The present study was undertaken to correlate the FNAC findings with histopathology so that rate of unnecessary thyoidectomies in benign pathologies should be avoided.

## 2. Material and Methods

This is a prospective analysis of seventy five consecutive patients of clinically diagnosed solitary thyroid nodule who presented to the Department of Surgery, Government Medical College Jammu, and Department of ENT, SMGS Hospital, Jammu (India) between January 2003 and December 2005.

All patients were evaluated by thorough clinical examination followed by routine investigations including haemogram, renal function tests, liver function tests, chest X-ray, lateral neck X-ray, thyroid function tests and, FNAC.

Inclusion criteria included clinically detected solitary thyroid nodule and euthyroid patients with normal thyroid function tests. Patients with abnormal thyroid function tests (hypothyroid/hyperthyroid) and multiple thyroid nodules were excluded from the study. FNAC was performed with 23 gauge needle, smears were fixed with ether-95% alcohol solution, and staining was performed using papanicolau's staining. After FNAC, all the patients were subjected to surgery after preoperative preparation and anaesthesia checkup. Thyroidectomy specimen was evaluated by histopathological examination. Specimens were processed in automated tissue processing units and staining was performed with routine haematoxylin and eosin stain.

Correlation of histopathological findings was performed with FNAC. Sensitivity, specificity, accuracy, positive predictive value, and negative predictive value were calculated for neoplastic and carcinomatous lesions.

## 3. Results

A total of 75 patients with solitary thyroid nodule were identified: 6 (8%) were male and 69 (92%) were females. Age of the patients ranged from 22 to 58 years with mean age of 38.7 years. Characteristics of the patients were shown in Table 1.

51 (68%) patients were from plain areas and 24 (32%) were residents of hilly areas. Commonest presentation was neck swelling in 60 (80%) of the patients. Duration of complaints ranged from six days to twenty years and mean duration was 1.7 years.

FNAC results revealed 39 (52%) cases as colloid nodular goitre, 12 (16%) as follicular neoplasm, 9 (12%) as papillary carcinoma, 6 (8%) as hurthle cell lesions, 6 (8%) as benign cystic lesions, and 3 (4%) cases as suspected of malignancy.

Histopathological examination of excised specimens showed 42 (56%) cases as colloid nodular goitre, 12 (16%) as follicular adenoma, 12 (16%) as papillary carcinoma, 3 (4%) as hurthle cell adenoma, 3 (4%) as hurthle cell changes with capsular invasion and, 3 (4%) as hashimoto's thyroiditis.

Comparison of FNAC with histopathological findings was performed. 45 cases were diagnosed as colloid nodular goitre and benign cystic lesions by FNAC. 39 of these cases were nonneoplastic lesions, 3 as papillary carcinoma and 3 as follicular adenoma in histopathological examination (Table 2).

30 cases were diagnosed as neoplastic lesions (follicular neoplasm, hurthle cell lesions, papillary carcinoma, and suspected malignancy) by FNAC. 3 of these cases were nonneoplastic lesions, 12 were benign neoplastic lesions, 12 were carcinoma, and 3 cases of suspected malignancy were diagnosed as hashimoto's thyroiditis on histopathological examination (Table 3).

False positive and false negative results were shown in Table 4.

Statistical analysis of neoplastic lesions (Table 5) showed sensitivity, specificity, accuracy, false positive rate, false negative rate, positive predictive value, and negative predictive value of FNAC to be 80%, 86.6%, 84%, 13.3%, 20%, 80%, and 86.6%, respectively whereas statistical analysis of carcinomatous lesions (Table 6) showed sensitivity, specificity, accuracy, false positive rate, false negative rate, positive predictive value, and negative predictive value of FNAC to be 80%, 95%, 92%, 5%, 20%, 80%, and 95%.

A total of 15 cases of solitary thyroid nodules were diagnosed as having malignant and the most common malignant lesion detected was papillary carcinoma, 12 out of 15 (80%).

## 4. Discussion

In present study, the age of patients ranged from 22 to 58 years with mean of 38.72 years. This age range and mean incidence is slightly lower as compared with previous studies [4–6]. We found that majority of patients (44%) were in their third decade of life. This is in accordance with the study by Dorairajan and Jayashree [7]. Solitary thyroid nodules were 4–9 times more common in females as compared to males [7, 8]. Our study showed that solitary thyroid nodules were 11 times more common in females than males.

The false negative rate was 20% in cases of neoplastic lesions. It constitutes a serious limitation of this technique since these malignant lesions would go untreated. The incidence of false negative results is as low as 1% to as high as 30% [9, 10]. The false positive rate was 13.3% for neoplastic lesions but none of these lesions were malignant.

Comparison of results of present study with various previous studies is shown in Table 7.

The methods used for the calculation of sensitivity, specificity, accuracy, positive predictive value, and negative predictive value were similar to previous studies [11, 12]. Sensitivity and accuracy of FNAC for detection of neoplasm were 80% and 84%, respectively, whereas they were 76% and 69%, respectively, in a study by Cusick et al. [12].

The sensitivity, specificity, and accuracy of FNAC for solitary thyroid nodules were 80%, 86.6%, and 84%, respectively, in our study whereas sensitivity, specificity, and accuracy of FNAC were 93.5%, 75%, and 79.6%, respectively, in a study by Bouvet et al. [8] and 79%, 98.5%, and 87%, respectively, in a study by Kessler et al. [13].

In our study 15 cases were found to be malignant on histopathological examination (12 papillary carcinoma and 3 hurthle cell lesions). It is to be stressed that all cases of papillary carcinoma diagnosed by FNAC were papillary carcinoma on histopathological examination also. This is in accordance with previous studies [7, 13]. The incidence of malignancy in this study was 20% which is in accordance with study by Dorairajan and Jayashree [7]. The incidence of malignancy can be as high as 43.6% [8].

The incidence of papillary carcinoma in the present study was 80%. In the literature, incidence of papillary carcinoma varies from 50% to 80% [7, 8, 14]. Brooks et al. found that preoperative FNAC had no direct impact on the selection of the surgical procedure and intraoperative frozen section added very little to surgical management [15].

The diagnostic accuracy of Correlation between FNAC diagnosis and final histological diagnosis, intraoperative frozen section diagnoses without intraoperative cytology and final histological diagnoses, and intraoperative frozen section diagnoses associated with intraoperative cytology and final histological diagnoses were 88.8%, 88.8%, and 95.7%, respectively [16].

Frozen section should be considered unnecessary because it does not affect the intraoperative decision making. The sensitivity, specificity, positive predictive value, negative predictive value, and accuracy were 13.0%, 97.3%, 37.5%, 90.0%, and 88.1% for FNA cytology, and 17.4%, 100%, 100%, 90.8%, and 91.0% for FS, respectively [17].

Analysis of data from seven series showed a false-negative rate of 1% to 11%, a false-positive rate of 1% to 8%, a sensitivity of 65% to 98%, and a specificity of 72% to 100% [18]. The results are consistent with our study.

Cytologic and histologic diagnoses were compared in 4069 patients and the sensitivity and specificity of FNAC were found to be 91.8% and 75.5%, respectively [19].

## 5. Conclusions

We concluded that FNAC diagnosis of malignancy is highly significant and such patients should be subjected to surgery. A benign FNAC diagnosis should be viewed with caution as false negative results do occur and these patients should be followed up and any clinical suspicion of malignancy even in the presence of benign FNAC requires surgery.

## Figures and Tables

**Table 1:** Characteristics of the patients presented with clinically solitary thyroid nodule.

Characteristic	Total patients (*n* = 75)
Age (in years)	
20–29	9
30–39	33
40–49	24
50–59	9

Sex	
Male	6
Female	69

Demography	
Plains	51
Mountains	24

Presenting complaint	
Neck swelling	60
Neck pain	9
Neck discomfort	6

Mode of detection of swelling	
Self	60
Others	15

Duration of complaints	
<1 month	3
1–12 months	24
1–2 Year	9
>2 years	39

Site of swelling	
Right lobe	45
Left lobe	21
Isthmus	9

Treatment history	
No	48
Yes	27

**Table 2:** Nonneoplastic lesions diagnosed by FNAC and their comparison with histopathological diagnosis.

FNAC report	Number of patients (*n* = 45)	Histopathological report	Number of patients (*n* = 45)	Remarks
Colloid nodular goitre & benign cystic lesions	45	Colloid nodular goitre	39	True negative
		Follicular adenoma	3	False negative
		Papillary carcinoma	3	False negative

**Table 3:** Benign or suspicious neoplastic lesions diagnosed by FNAC and their comparison with histopathological diagnosis.

FNAC report	Number of patients (*n* = 30)	Histopathological report	Number of patients (*n* = 30)	Remarks
Follicular neoplasm	12	Follicular adenoma	9	True positive
		Colloid nodular goitre	3	False positive
Hurthle cell tumours	6	Hurthle cell adenoma	3	True positive
		Hurthle cell carcinoma	3	True positive
Papillary carcinoma	9	Papillary carcinoma	9	True positive
Suspected malignancy	3	Hashimoto thyroiditis	3	False positive

**Table 4:** Summary of false positive and false negative results of FNAC.

FNAC finding	Histopathology result
False positive	
Follicular neoplasm	Colloid nodular goitre
Suspected malignancy	Hashimoto's thyroiditis

False negative	
Colloid nodular goitre	Follicular adenoma
Colloid nodular goitre	Papillary carcinoma

**Table 5:** Statistical analysis for neoplastic lesions.

Test being evaluated (FNAC)	Reference standard test (Histopathology)	
	Positive	Negative
Positive + suspicious	24	6
Negative	6	39

Sensitivity = 80%, specificity = 86.6%, accuracy = 84%, false positive result = 13.3%, false negative result = 20%, positive predictive value = 80%, and negative predictive value = 86.6%.

**Table 6:** Statistical analysis for carcinomatous lesions.

Test being evaluated (FNAC)	Reference standard test (Histopathology)	
	Positive	Negative
Positive + suspicious	12	3
Negative	3	57

Sensitivity = 80%, specificity = 95%, accuracy = 92%, false positive result = 5%, false negative result = 20%, positive predictive value = 80%, and negative predictive value = 95%.

**Table 7:** Comparison of results of present study with previous studies.

Study	Year	Number of patients	Sensitivity	Specificity	Accuracy	Negative predictive value	Positive predictive value
Al-Sayer et al.	1985	70	86	93	92	96	80
Cusick et al.	1990	283	76	58	69	64	72
Bouvet et al.	1992	78	93.5	75	79.6	88.2	85.3
Afroze et al.	2002	170	61.9	99.3	94.5	94.7	92.8
KO HM et al.	2003	207	78.4	98.2	84.4	66.3	99
Kessler et al.	2005	170	79	98.5	87	76.6	98.7
Present series	2006	75	80	86.6	84	86.6	80
